# Effect of mandibular advancement device treatment on HIF-1α, EPO and VEGF in the myocardium of obstructive sleep apnea–hypopnea syndrome rabbits

**DOI:** 10.1038/s41598-020-70238-0

**Published:** 2020-08-06

**Authors:** Dechao Zhu, Wenjing Kang, Shilong Zhang, Xing Qiao, Jie Liu, Chunyan Liu, Haiyan Lu

**Affiliations:** Department of Orthodontics, School and Hospital of Stomatology, Hebei Medical University & Hebei Key Laboratory of Stomatology, No. 383, East Zhongshan Road, Shijiazhuang, 050017 Hebei People’s Republic of China

**Keywords:** Anatomy, Biomarkers, Cardiology, Diseases, Pathogenesis, Risk factors, Signs and symptoms

## Abstract

The aim of this study was to investigate the effects of mandibular advancement device (MAD) therapy for obstructive sleep apnea–hypopnea syndrome (OSAHS) on hypoxia-inducible factor-1α (HIF-1α), erythropoietin (EPO) and vascular endothelial growth factor (VEGF) in myocardial tissue. New Zealand rabbits were used to develop OSAHS and MAD models. Cone beam computed tomography (CBCT) of the upper airway and polysomnography (PSG) recordings were performed with the animals in the supine position. All of the animals were induced to sleep in a supine position for 4–6 h each day and were observed continuously for 8 weeks. The myocardial tissue of the three groups was dissected to measure the expression of HIF-1α, EPO and VEGF. The results showed that there was higher expression of HIF-1α, EPO and VEGF in the OSAHS group than those in the MAD and control groups. MAD treatment significantly downregulated the expression of HIF-1α, EPO and VEGF in the OSAHS animals. We concluded that MAD treatment could significantly downregulate the increased expression of HIF-1α, EPO and VEGF in OSAHS rabbits, improving their myocardial function.

## Introduction

Obstructive sleep apnea–hypopnea syndrome (OSAHS) is an increasingly common public health problem, with a prevalence of 9% of the middle-aged male population and 4% of the female population^1^. The stenoses or occlusion of the upper airway during sleep can cause repeated airflow cessation and reduction, snoring and daytime sleepiness. Increasing amounts of evidence have shown that OSAHS is an independent risk factor for cardiac hypertrophy, coronary atherosclerosis, thrombus and heart failure^2–4^. As a crucial factor, hypoxia-inducible factor-1α (HIF-1α) plays an important role in the hypoxia adaptive pathway, which is regulated by the oxygen content in cells and tissues^5^. Erythropoietin (EPO) and vascular endothelial growth factor (VEGF), the downstream factors of HIF-1α, are involved in erythropoiesis, angiogenesis, the regulation of extracellular matrix and apoptosis, affecting cardiac function and morphology^6–9^. However, little is known about the changes in HIF-1α, EPO and VEGF in the myocardium of OSAHS patients.

Mandibular advancement devices (MAD) have been widely applied for patients with mild to moderate OSAHS or are used as a substitute for treating severe OSAHS due to being convenient, affordable and having fewer side effects than continuous positive airway pressure (CPAP)^10,11^. Most studies have focused on the changes in the subjective symptoms and airway size of OSAHS patients after MAD treatment. However, its effect on hypoxia adaptive pathways is still controversial. This study was designed to evaluate the changes of HIF-1α, EPO and VEGF in the myocardium of OSAHS animal models we developed previously^12^ and to further investigate the effects of MAD treatment on these changes, providing evidence to guide clinical treatment.

## Materials and methods

All methods in this study were performed in accordance with the medical ethics committee in Hospital of Stomatology, Hebei Medical University. Animal use and care was in accordance with the guidelines of the medical ethics committee for the housing and care of animals bred, supplied and used for scientific purposes. All experiments were performed in accordance with relevant guidelines and regulations. The work was approved by the medical ethics committee in Hospital of Stomatology, Hebei Medical University (Certificate No. [2017]016).

### Animal model development

**Figure 1:**
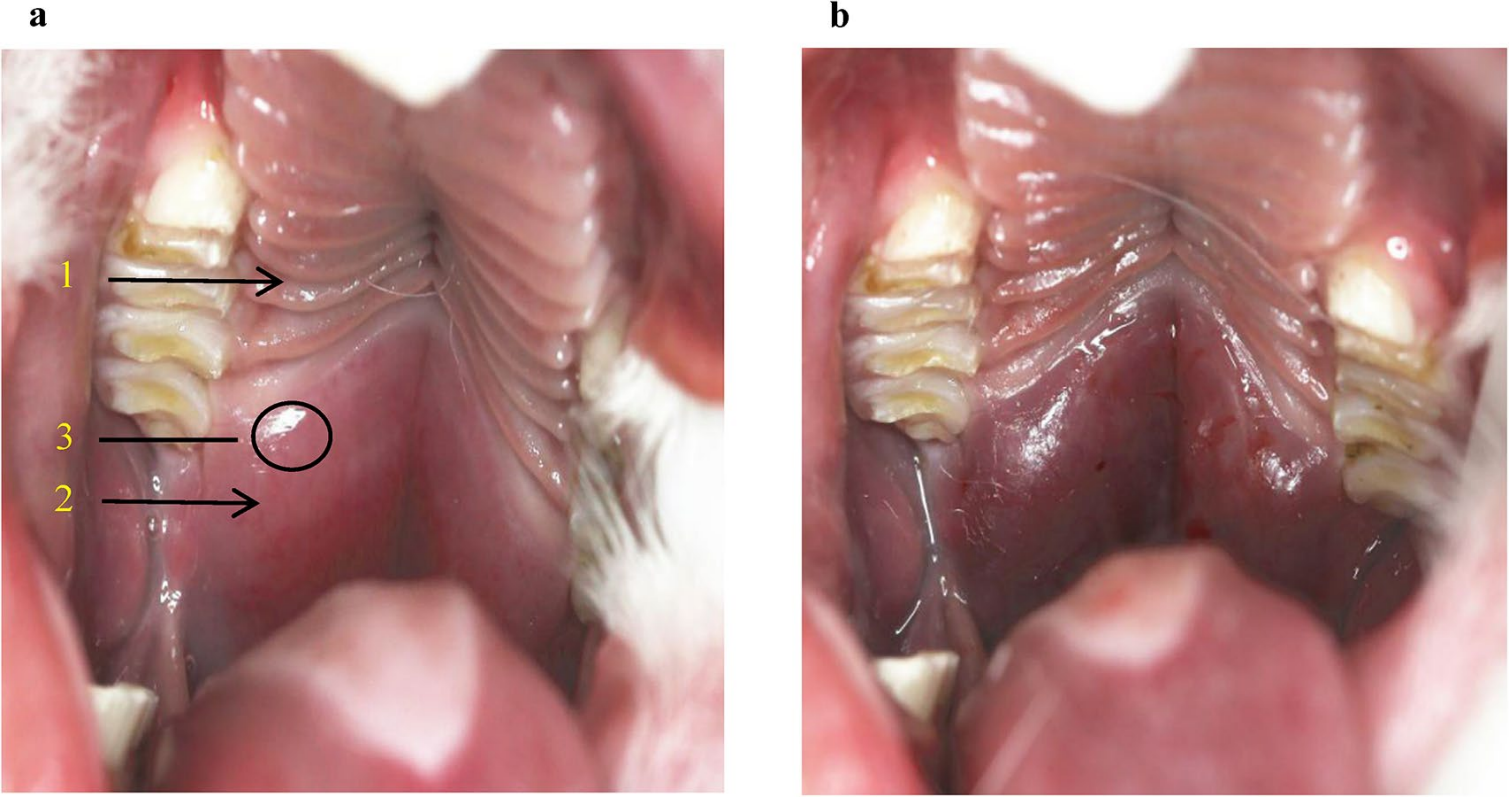
Injection point. Arrow 1 and 2 show the hard palate and soft palate, respectively, and circle 3 shows the injection point, which is 1.5 cm away from the junction of the soft and hard palate (**a**). Then, 2 ml of medical sodium hyaluronate gel was injected into the submucous muscular layer (**b**).

Every effort was made to minimize animal pain and suffering. A total of 18 male 6-month-old New Zealand white rabbits (initial weight, 3–3.5 kg) were randomly divided into three groups: the OSHAS, MAD, and control groups, with 6 rabbits in each group. All of the animals were housed under normal laboratory conditions. Food and water were available ad libitum. The animal models were developed in the same way as in our previous studies^12,13^. Briefly, OSAHS was induced via injection of gel into the submucous muscular layer in the centre of the soft palate 1.5 cm away from the junction of the soft and hard palate (Fig. 1). No gel was injected in the control group. Animals in these two groups were later confirmed to have OSAHS by clinical signs, CBCT scanning and polysomnography (PSG).

### Three-dimensional model reconstruction and polysomnography (PSG)

The images of the upper airway structure within the range from the cranial crest to the clavicle were obtained by CBCT scanner (KaVo 3D eXam, USA): a single 360° rotation scan, 120 kV voltage, 5 mA current, slice thickness 0.3 mm, scanning time 17.8 s. The midpoint cross line of the interpupil line overlapped with the central cross line of the location line during scanning. All of the images were imported into Mimics version 21.0 (Materialise Inc., Belgium. www.materialise.com) and then upper airway three-dimensional models were rebuilt. The volume, cross-sectional areas, sagittal diameter and cross diameter from the top level of the soft palate to the level of 1/4, 2/4 and 3/4 in the posterior airway were measured. PSG monitoring was performed as previously described^12^ (Fig. S1).

### Treatment with a mandibular advancement device

**Figure 2:**
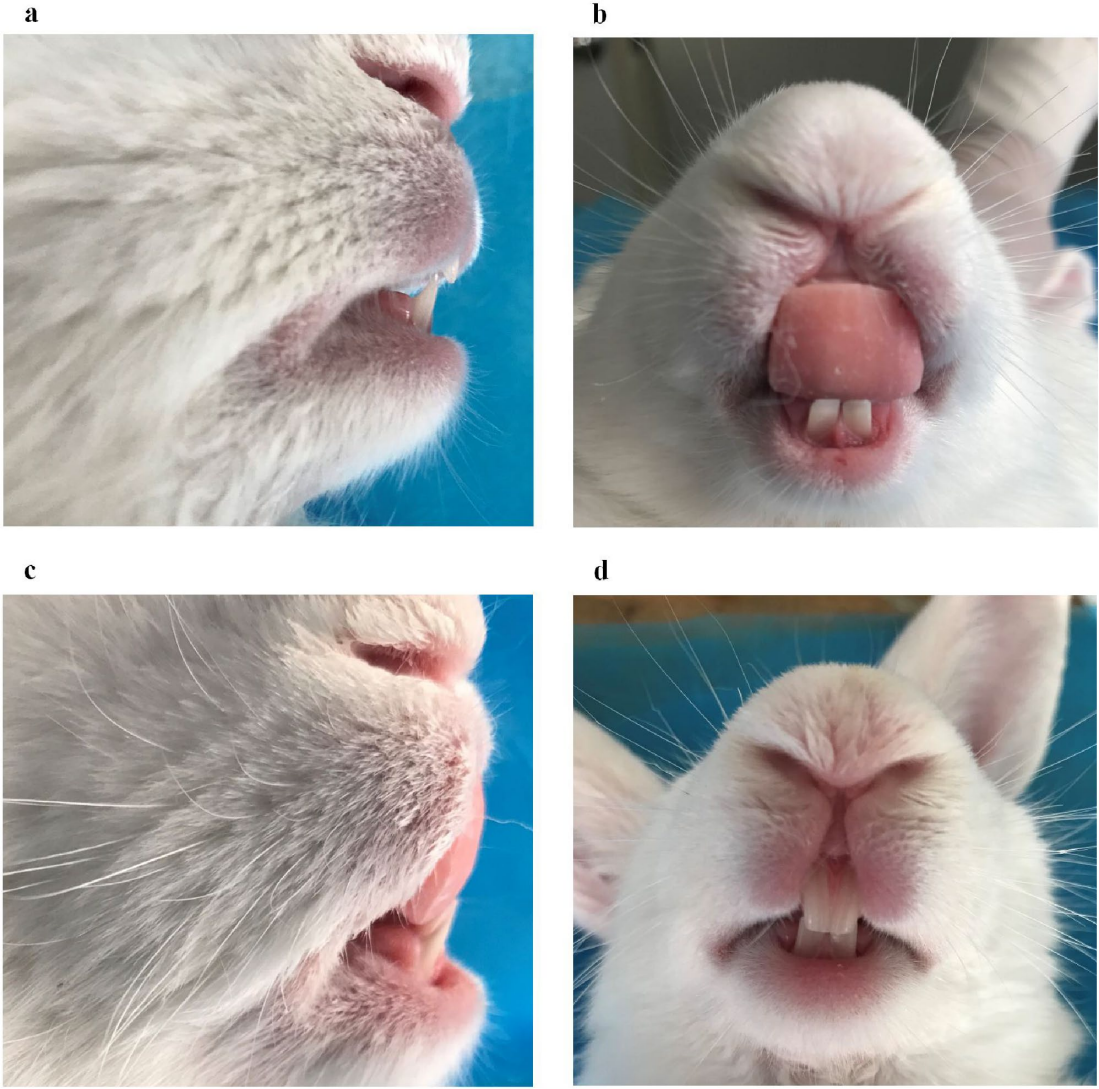
The pictures before (**a**,**b**), and after MAD treatment; the mandible was guided forward 3–4 mm with the MAD inserted (**c**,**d**).

MADs were made of self-curing composite resin and adhered to the two upper incisors with glass ionomer (3M ESPE AG, Seefeld, Germany). The mandible was advanced forward 3–4 mm by the MAD with a 30° inclined plane on the incisors (Fig. 2). CBCT scanning was performed to evaluate the effectiveness of the MADs. After group OSAHS and group MAD were both induced successfully, all animals were orally perfused with 10% chloral hydrate at a dose of 5–6 ml/kg to induce sleeping. They were then trained to sleep independently in a supine position in a homemade wooden box. All of the rabbits were forced to sleep supine for 8 weeks (Fig. S2).

### Western blot

After 8 weeks, the myocardial tissue was collected and cryopreserved at − 80 °C. HIF-1α protein expression was determined by using Western blots. Myocardial nucleus proteins were extracted according to the manufacturer’s protocol (Kang Wei Century Biotechnology Co., Beijing). The protein concentration was determined by using a bicinchoninic acid assay. Protein samples (75 μg) were electrophoresed on polyacrylamide gels (10%). After the proteins were transferred to PVDF membranes, the samples were blocked in a solution of 5% skim milk, incubated at 4 °C overnight with primary antibodies against HIF-1α (1:1,000; BD, USA) and glyceraldehyde-3-phosphate dehydrogenase (GAPDH; 1:5,000; Sungene Biotech, Tianjin, China), and then incubated with the secondary antibody, goat anti-rabbit IgG/HRP (Bio Basic, USA, 1:5,000) for 40 min, and then the blots were developed by enhanced chemiluminescence after a 5-min exposure. A Quantity One image analytical system (Bio-Rad, USA) was used for analysing the intensity of the protein bands. The accumulation of HIF-1α was quantified as the ratio of the band intensities for the target protein and GAPDH.

### Real-time PCR

Samples of the myocardial tissue from the rabbits in the three groups were homogenized in TRIzol. Total RNA was extracted from the myocardial tissues, and real-time PCR was performed with a Quant Gene 9600 Real-Time PCR System (BIOER, Japan). The relative levels of expression of the targeted molecules were calculated by an Exicycler 96 PCR System (Bioneer, Alameda, CA, USA) that used the 2-ΔΔCt method. The expression levels were normalized to GAPDH, which served as an internal reference. The sequences of the primer pairs are listed in Table 1.

**Table 1:** Real-time PCR primers used in this study.

Genes	Forward primer 5′–3'	Reverse primer 5′–3'	Length (bp)
HIF-1α	5′CGACTTCCAGTTGCGGTCCTTC 3'	5′CGGTGTTGGCGGCAGGTTC 3'	110
GAPDH	5′GAAGGTCGGAGTGAACGGA 3'	5′ACTCGCTCCTGGAAGATGG 3'	290

### Enzyme-linked immunosorbent assay (ELISA)

Myocardial tissue (100 mg) from the three groups was homogenized. After centrifugation at 8,000 r/min, ELISA was applied to the supernatant according to the manufacturer’s instructions (Colorfulgene, Wuhan, China). The accumulation of EPO and VEGF was quantified using optical density with a spectrophotometer (NanoDrop, USA).

### Statistical analysis

All data were analysed in the Statistical Package for SPSS version 21.0 (SPSS Inc., Chicago, Illinois, USA) and expressed as the mean ± SD. The data were tested for normality and homogeneity of variance. Data with a normal distribution and variance homogeneity were compared with single factor ANOVA, an LSD test was used for multi-group mean pairwise comparisons, a rank sum test was used for non-normal distributions, and the LSD test was used for multi-group mean pairwise comparisons. A statistically significant difference was defined as *P* < 0.05.

## Results

### OSAHS-like clinical symptoms could be relieved by MAD treatment

In the control group, there was uniform sleep respiration, and no snoring was detected in the supine position. However, OSAHS-like symptoms such as snoring, awakening and enhancement of thoracoabdominal movement occurred repeatedly in the supine position in the OSAHS group. Sleep respiration in the MAD group was basically uniform, and the above OSAHS-like symptoms were significantly reduced, which suggested that the MADs could relieve clinical symptoms induced by upper airway obstruction.

### The narrowed upper airway could be corrected by MAD treatment

**Figure 3:**
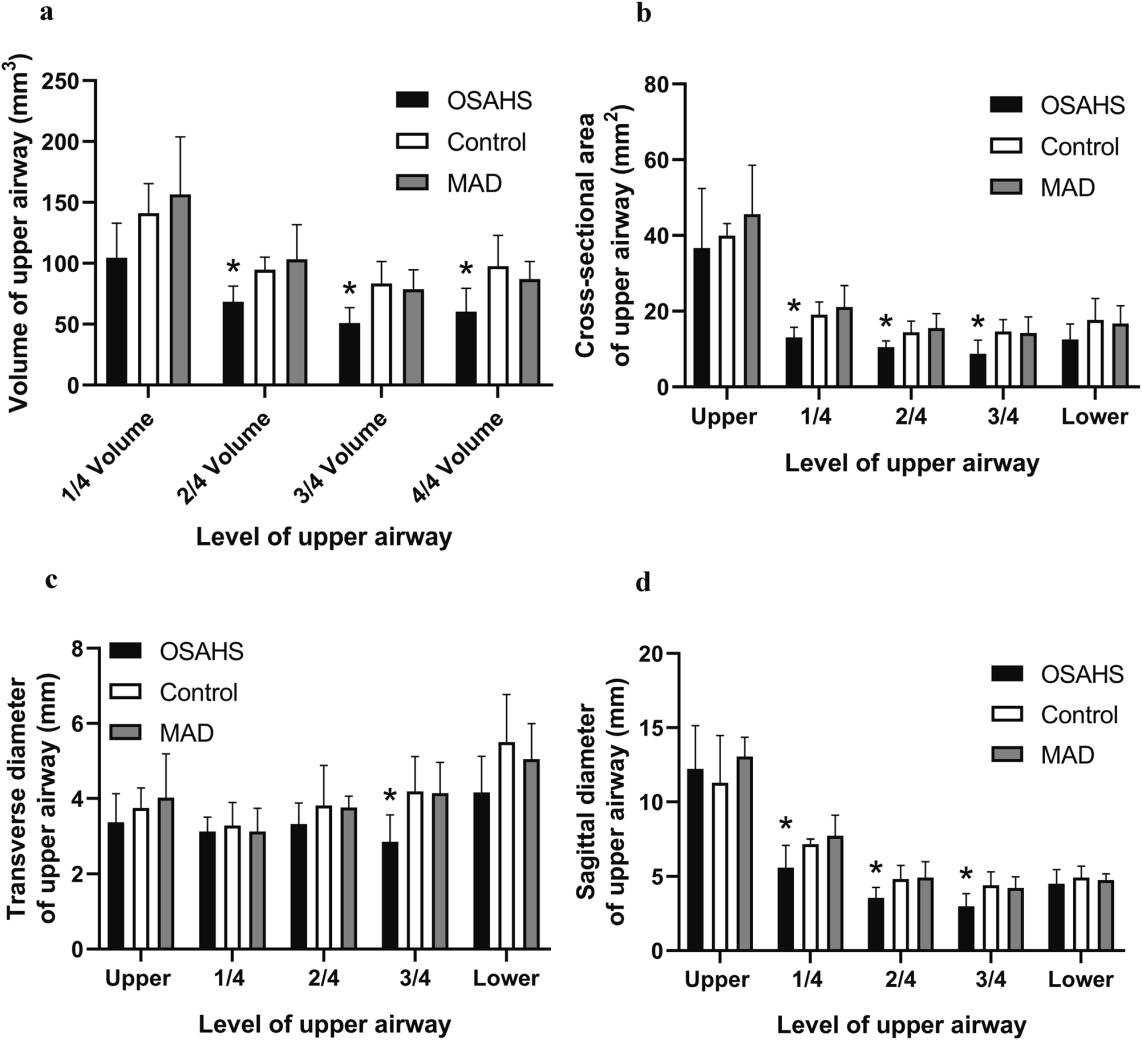
The volume, cross-sectional area, transverse diameter and sagittal diameter at each level of the upper airway (**a**–**d**). All data were obtained from three biologically independent experiments. The results are expressed as mean ± SD. Statistically significant differences are indicated by an asterisk; *P < 0.05; *OSAHS* obstructive sleep apnea–hypopnea syndrome, *MAD* mandibular advancement device.

**Figure 4:**
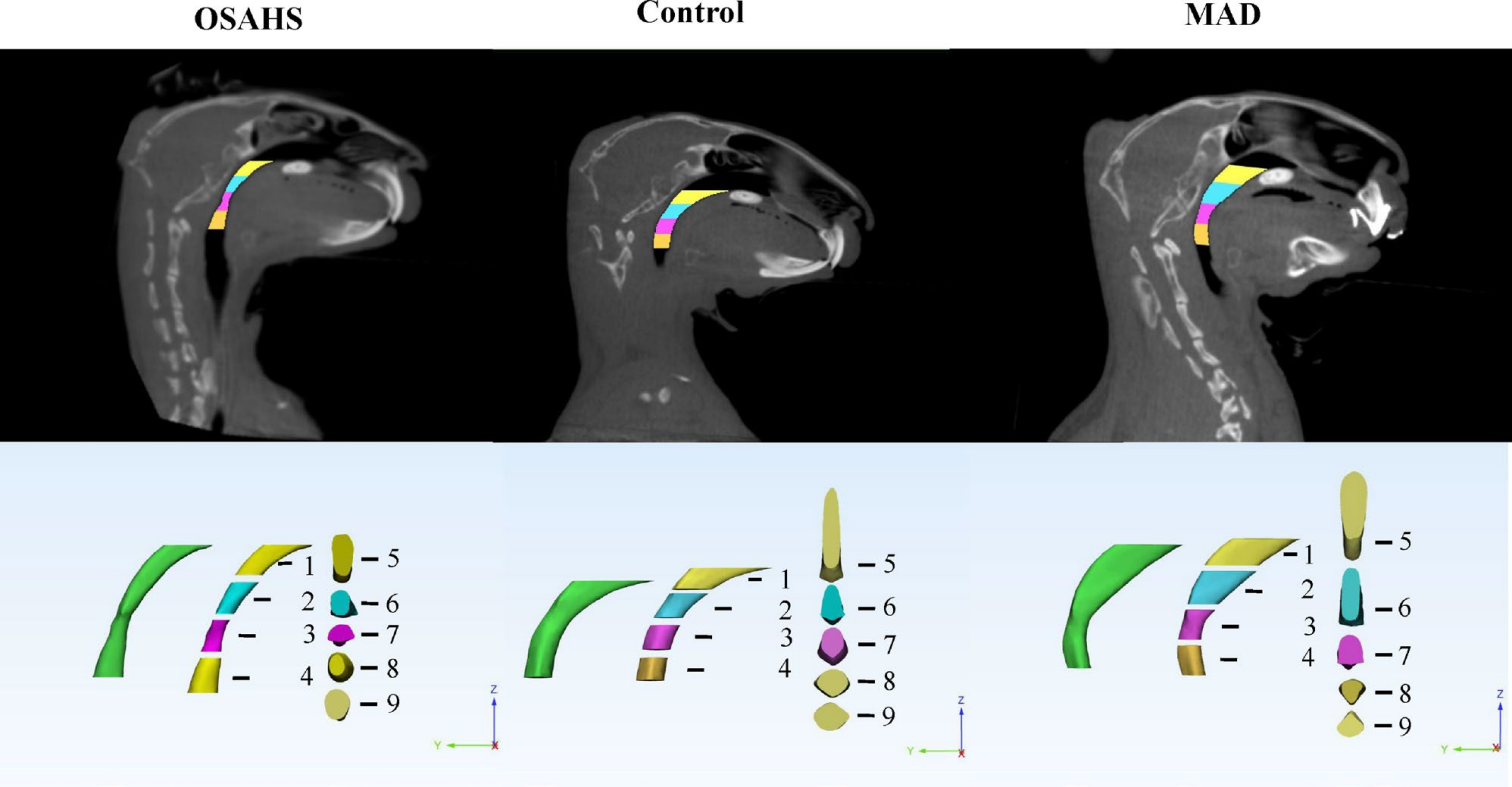
Three-dimensional reconstruction models of the upper airway of the OSAHS, MAD, and control groups. All data were obtained from three independent experiments. 1: 1/4 Volume; 2: 2/4 Volume; 3: 3/4 Volume; 4: 4/4 Volume; 5: Upper Cross-sectional area, Upper Transverse diameter, Upper Sagittal diameter; 6: 1/4 Cross-sectional area, 1/4 Transverse diameter, 1/4 Sagittal diameter; 7: 2/4 Cross-sectional area, 2/4 Transverse diameter, 2/4 Sagittal diameter; 8: 3/4 Cross-sectional area, 3/4 Transverse diameter, 3/4 Sagittal diameter; 9: Lower Cross-sectional area, Lower Transverse diameter, Lower Sagittal diameter.

The rabbits in the OSAHS group presented with a narrower upper airway, such as a significantly reduced volume, cross-sectional area, and sagittal diameter at different levels (*P* < 0.05), The upper airway was enlarged to the normal level in the MAD group, similar to the control group (*P* > 0.05; Figs. 3, 4 and Table S3).

### The respiration parameters could be rescued by MAD treatment

**Figure 5:**
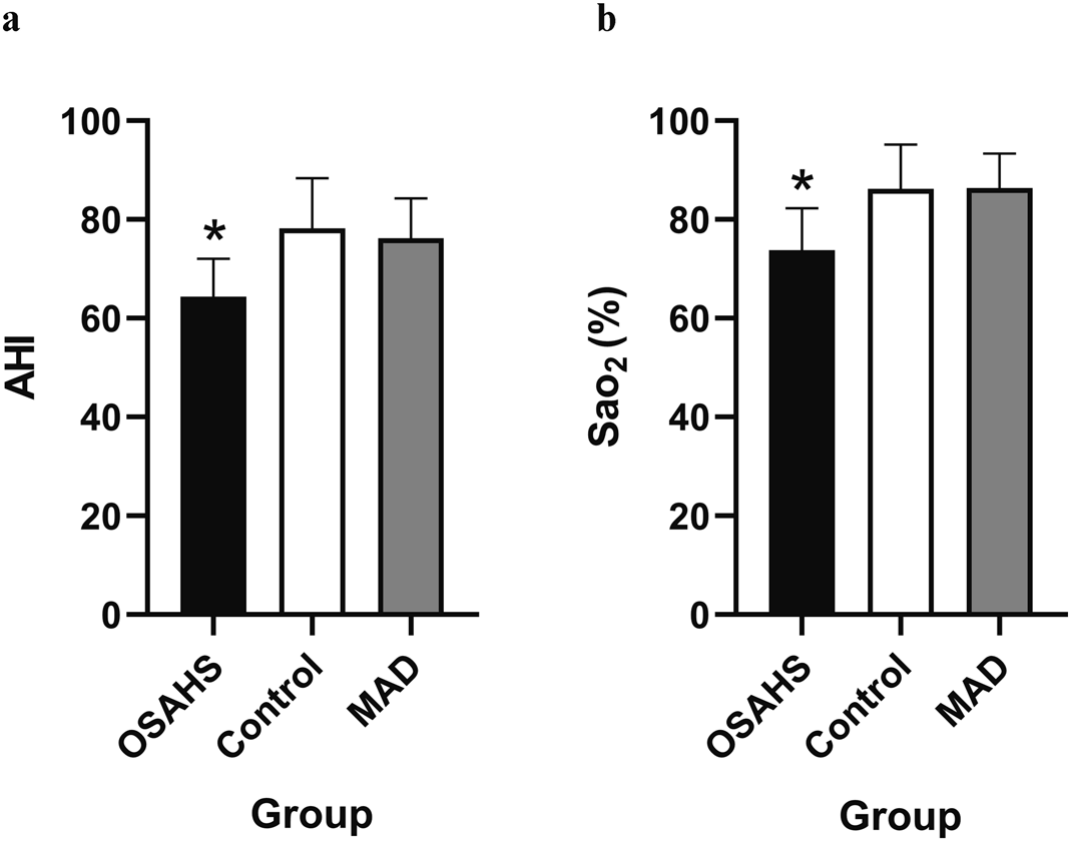
The AHI and Sao_2_% were recorded by PSG (**a**,**b**). All data were obtained from three biologically independent experiments. The results are expressed in mean ± SD. Statistically significant differences are indicated by an asterisk; *P < 0.05; *OSAHS* obstructive sleep apnea–hypopnea syndrome, *MAD* mandibular advancement device, *AHI* apnea–hypopnea index, *Sao*_*2*_*%* oxygen saturation.

**Figure 6:**
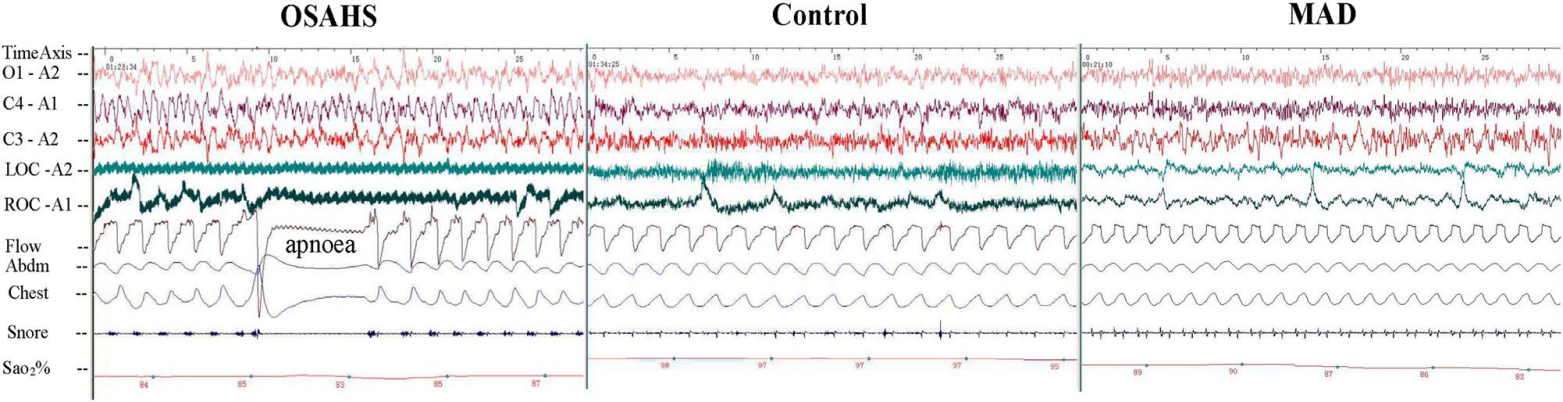
The polysomnography records for the OSAHS, MAD, and control groups. Symptoms of apnoea developed in the OSAHS group.

There was significantly increased AHI and significantly decreased average oxygen saturation (SaO_2_%) in the OSAHS group compared with that in the control group (*P* < 0.05). According to the definitions of hypopnea, apnoea, and AHI mentioned above, rabbits in the OSAHS and MAD groups were confirmed to have OSAHS during sleep. With MAD treatment, the respiration parameters became normal and similar to those in the control group (*P* > 0.05; Figs. 5, 6 and Table S4).

### The increased protein level of HIF-1α in the OSAHS group was downregulated by MAD treatment

**Figure 7:**
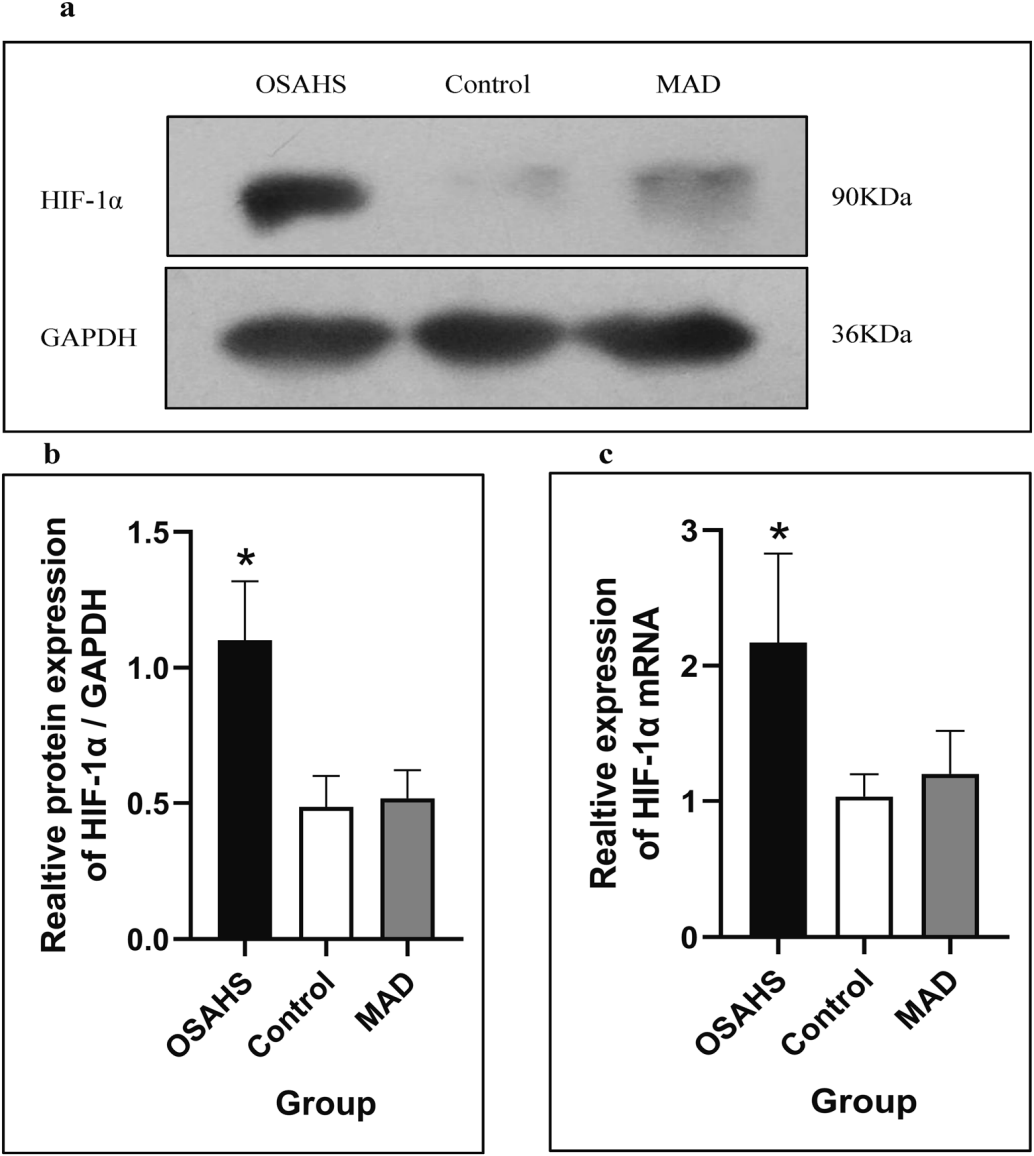
The western blot analysis of HIF-1α in the three groups (**a**) and the expression of HIF-1α and the relative expression of HIF-1α mRNA (**b**,**c**). All data were obtained from three biologically independent experiments. The results are expressed as mean ± SD. Statistically significant differences are indicated by an asterisk; *P < 0.05; *OSAHS* obstructive sleep apnea–hypopnea syndrome, *MAD* mandibular advancement device, *HIF-1α*, hypoxia-inducible factor-1α, *GAPDH* glyceraldehyde-3-phosphate dehydrogenase. Full-length blot images are shown in the Supplementary Information (Fig. S14).

The relative protein levels of HIF-1α in the three groups are shown in Fig. 7. The expression level of HIF-1α was significantly higher in the OSAHS group than in the control group (*P* < 0.05). There was no significant difference between the MAD and control groups (*P* > 0.05; Fig. 7a,b, Table S5 and Fig. S6).

There was a significant increase in the expression of HIF-1α mRNA in the OSAHS group compared with that in the control group (*P* < 0.05), but there was no significant difference between the MAD and control groups (*P* > 0.05; Fig. 7c, Table S7 and Fig. S8–Fig. S110).

**Figure 8:**
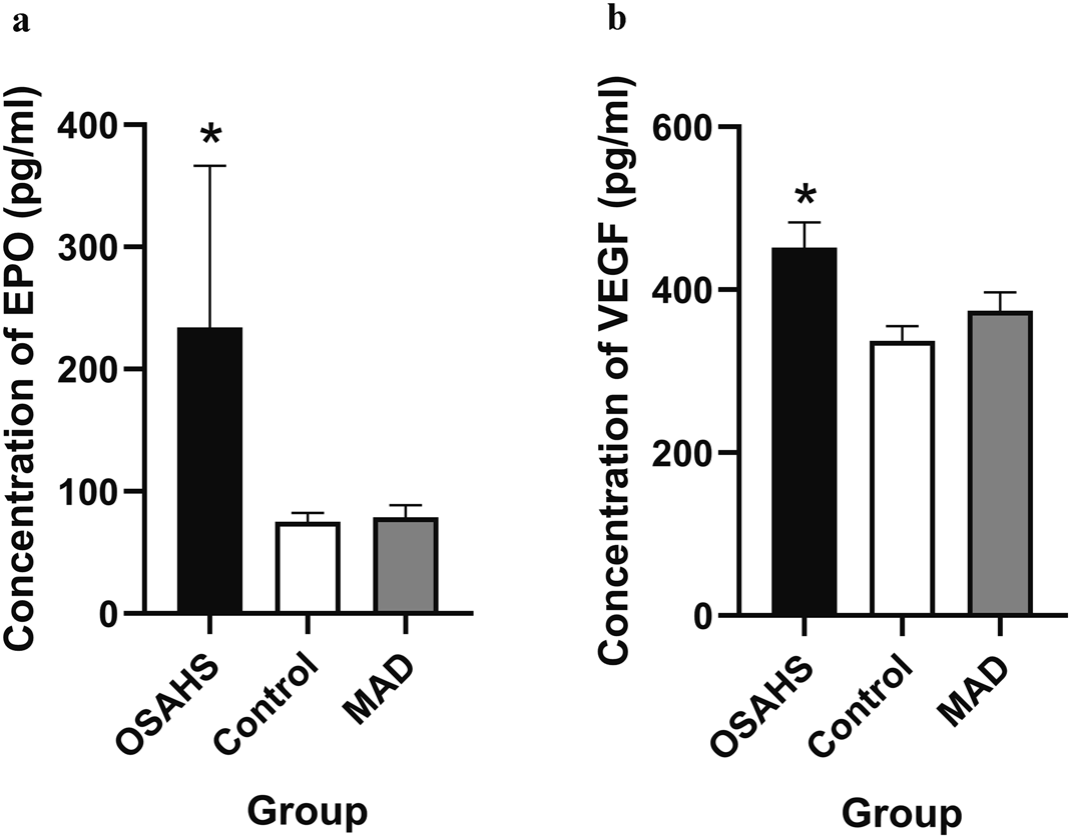
Concentrations of EPO and VEGF are shown in (**a**,**b**). All data were obtained from three biologically independent experiments. The results are expressed as the mean ± SD. Statistically significant differences are indicated by an asterisk; *P < 0.05; *OSAHS* obstructive sleep apnoea–hypopnea syndrome, *MAD* mandibular advancement device, *EPO* erythropoietin, *VEGF* vascular endothelial growth factor.

The ELISA results are shown in Fig. 8, Table S11, Fig. S12 and Fig. S13. There were significantly higher levels of EPO and VEGF in the OSAHS group than in the MAD and control groups (*P* < 0.05). In contrast, there was no significant difference between the MAD and control groups (*P* > 0.05).

## Discussion

The characteristics of obstructive sleep apnoea–hypopnea syndrome (OSAHS) are repeated partial or complete collapse of the pharyngeal cavity in the upper airway during the sleep process because of abnormal morphology of the upper airway, such as a disorder of myoelectric activity, hypertrophy of a gland, obesity, and the body position^14^.

The traditional method for studying the structure of the upper airway is measurement of the X-ray cephalometric. Previous studies have confirmed the existence of a stenosis in the sagittal direction of the upper airway in OSAHS patients^15,16^. However, this method can only be used for two-dimensional plane measurements. The three-dimensional changes of the upper airway are still unclear. Therefore, CBCT scanning and three-dimensional reconstruction were used in this study. One of the advantages of three-dimensional measurements of the complex structure of the upper airway is that they are more precise and objective^17,18^.

In this study, we found that the upper airway stenosis of OSAHS was located in the palatopharynx and glossopharynx and that the volume, cross sectional area and sagittal diameter became significantly smaller. However, there were no significant changes at the transverse level. Meanwhile, the AHI increased and the Sao_2_% decreased significantly in the OSAHS group. The above results indicate that we successfully established an OSAHS model. Moreover, the location of the upper airway stenosis is similar to the results of many previous studies^19,20^. Previous research has confirmed that OSHAS is highly correlated with the occurrence and development of hypertension and vascular endothelial function injury^21,22^. However, the effect of HIF-1α expression on cardiovascular function in patients with OSHAS is still controversial. Our study confirmed that the expression of HIF-1α protein and mRNA in the myocardial nucleus was significantly increased in the OSAHS group. These results suggested that OSAHS could activate the oxidative stress pathway in myocardial tissue, with HIF-1α as the key mediator. When cardiomyocytes were in a hypoxic state, the hydrolysis of HIF-1α was inhibited and its expression was increased. Then, it entered the nucleus in the form of a dimer and regulated the transcription of downstream factors. In the long run, this may cause damage to the cardiovascular system^23–25^.

Under the condition of normal oxygen, the ubiquitin-mediated pathway is activated by the oxygen-dependent degradation domain (ODD), and the α subunit is ubiquitinated and hydroxylated on the prolyl residues^26^. Then, it binds to the von Hippel-Lindau tumour suppressor protein to form a specific recognition module to initiate the hydrolysis of HIF-1α protein. This is why it is difficult to detect HIF-1α in cells under normal conditions. However, in a hypoxic environment, the levels of ubiquitin and hydroxylation are decreased, and the degradation of HIF-1α is inhibited. HIF-1α binds to HIF-1β to form a stable heterogeneous dimer structure and then transfers from the cytoplasm to the nucleus and binds to the hypoxia response element (HRE) on target genes such as EPO and VEGF to initiate the transcription process^27,28^.

EPO and VEGF are downstream genes of HIF-1α that regulate and change physiological functions^29,30^. In this study, we found that the expression level of HIF-1α was increased, and the expression levels of EPO and VEGF were also increased. The possible mechanism may be that hypoxia activated the oxidative stress pathway and increased the expression of HIF-1α. It then promoted an increase of EPO and VEGF by combining with HREs^31,32^. EPO regulates the proliferation and differentiation of red blood cells, and can protect myocardial tissue against short-term anoxia^33^. However, under conditions of long-term hypoxia, the excessive proliferation of red blood cells may cause thrombosis formation^34^. VEGF can regulate the proliferation of vascular endothelial cells and maintain the stability of cardiovascular function under hypoxia. In addition, it can compensate for anoxic myocardial tissue by establishing new collateral circulation^35^. However, under conditions of long-term hypoxia, the vascular endothelial function becomes unbalanced. The function of the new vasculature is not stable enough, which leads to thickening of the cardiovascular wall, coronary atherosclerosis and myocardial hypertrophy.

Since the 1980s, a variety of mandibular extension appliances have been used for the treatment of OSAHS. One of the clinical case statistics of Lindman indicated that the efficacy rate of an oral appliance was approximately 61%^36^. Studies have confirmed that MADs can improve the shape of the stenosis, but the exact location of the stenosis is still controversial^37^. Therapy with MAD can improve cardiac function. In this study, we found that MAD enlarged the volume, cross sectional area and sagittal diameter of the palatopharynx and glossopharynx. AHI and blood oxygen saturation were also significantly improved after an MAD was inserted. These findings suggest that MADs can improve the symptoms of OSAHS by expanding the upper airway and increasing ventilation. In addition, the hypoxia status was improved, and the expression levels of HIF-1α, EPO and VEGF were significantly decreased, which indicates that MADs can expand the airway morphology and improve cardiac function.

In summary, the hypoxia adaptive pathway was activated in the OSAHS rabbit myocardium, which could promote the expression of HIF-1α and its downstream factors EPO and VEGF. MAD therapy could move the mandible forward and expand the airway to reduce the expression of HIF-1α, EPO and VEGF.

## Supplementary information

Supplementary file1 (DOC 10398 kb)
