# Supplementary material for: Effect of mandibular advancement device treatment on HIF-1α, EPO and VEGF in the myocardium of obstructive sleep apnea–hypopnea syndrome rabbits
Source: Sci Rep. 2020 Aug 6;10:13261. doi: 10.1038/s41598-020-70238-0 (PMC7414037; doi:10.1038/s41598-020-70238-0)
Supplement: Supplementary file 1 — Supplementary file1 (DOC 10398 kb) [file 41598_2020_70238_MOESM1_ESM.doc]

Effect of mandibular advancement device treatment on HIF-1α, EPO and VEGF in the myocardium of obstructive sleep apnea-hypopnea syndrome rabbits

Dechao Zhu^1^, Wenjing Kang^1^, Shilong Zhang^1^, Xing Qiao^1^, Jie Liu^1^,

Chunyan Liu^1*^ & Haiyan Lu^1*^

^1^ Department of Orthodontics, School and Hospital of Stomatology, Hebei Medical University & Hebei Key Laboratory of Stomatology, Shijiazhuang, 050017, P.R. China.

^*^ These authors have contributed equally to this paper.

^*^Correspondence to:

Chunyan Liu, E-mail: [liuchunyan0313@163.com;](mailto:liuchunyan0313@163.com;)

Haiyan Lu, E-mail: luhaiyan67@163.com.

No. 383, East Zhongshan Road, Shijiazhuang, Hebei 050017, P.R. China.


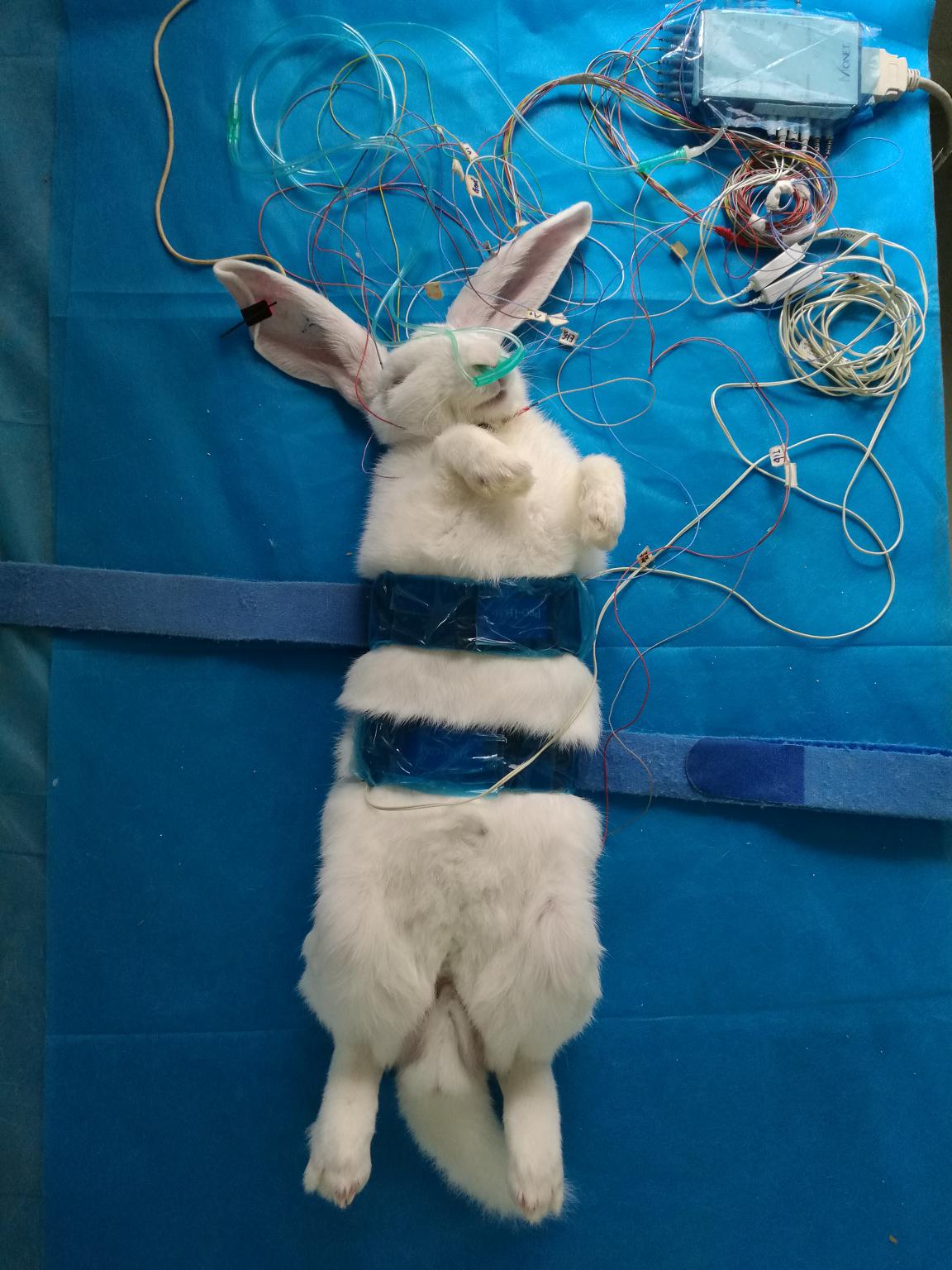

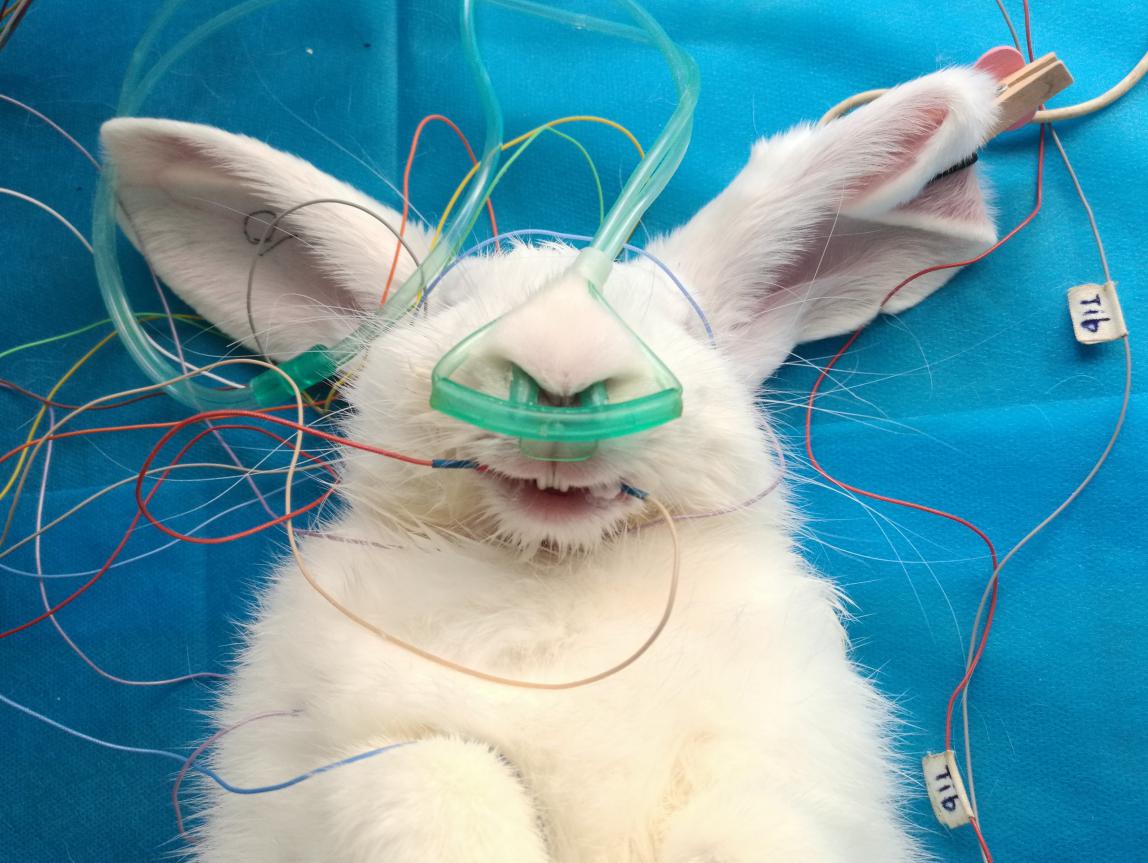

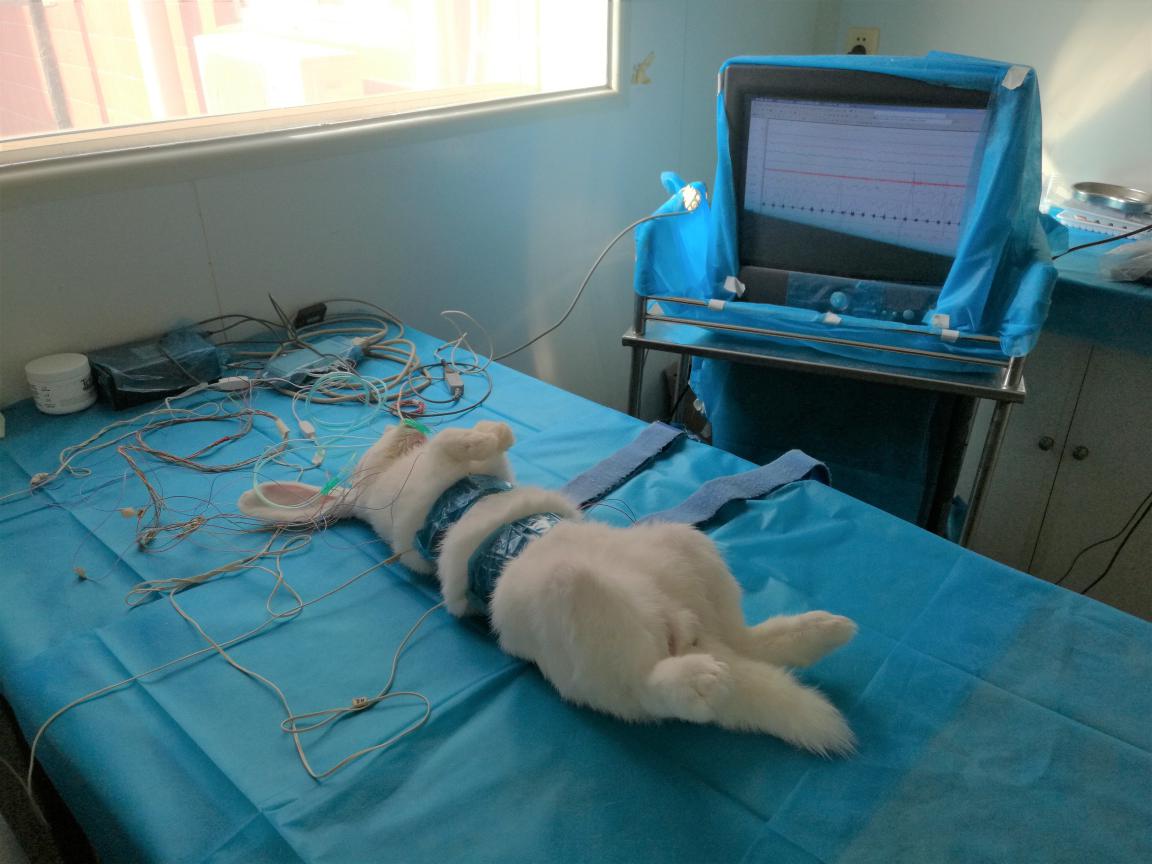


**Figure S1.** polysomnography (PSG) during sleeping in suspine. Barbering and cleaning electrodes. Connecting the EEG signals (C3/A2, C4/A1 and O1/A2) to crown; Connecting the LOC (left eye electricity) and ROC (right eye electricity) to eyes; EKGs was connected to the supraclavicular fossa on the right, the left side was connected to the myocardium-- the beating of the heart was the most obvious; Nose air flow catheter was fixed with white tape; The sensor of blood oxygen saturation was fixed at the middle ear artery. Monitoring was performed 2-4h, constantly. The data were analyzed to evaluate the success of OSAHS modeling and the efficacy of MAD in expanding narrow upper airway.


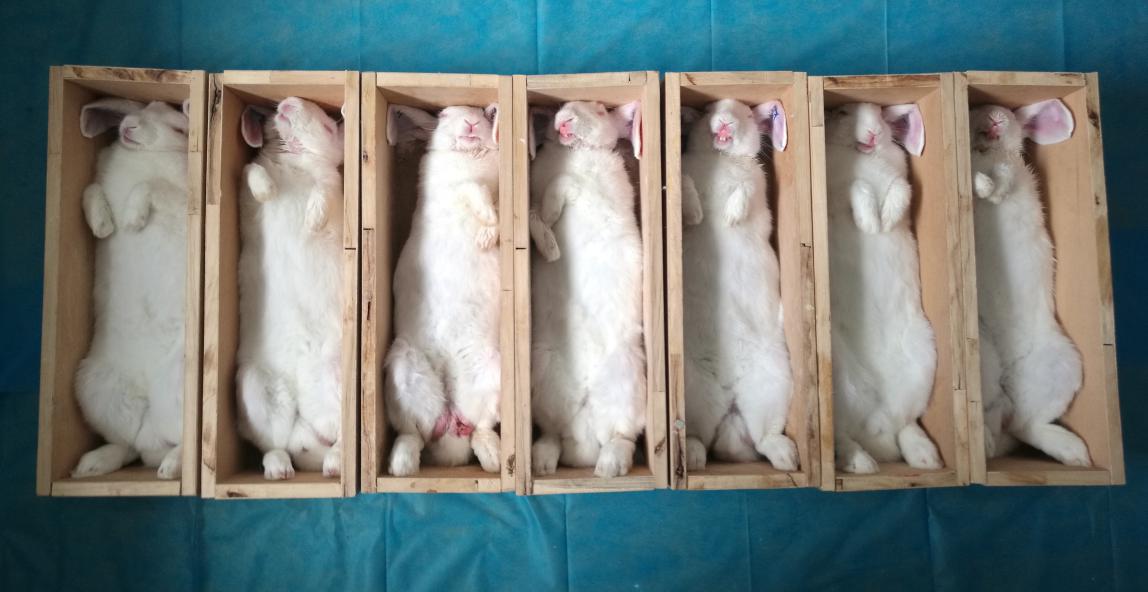


**Figure S2.** Sleeping in suspine. After modeling, the diet of the experimental animals was normal and there was no ulceration or infection in the injection area. The three groups of experimental animals were weighed daily and perfused with 10% chloral hydrate through oral cavity at a dose of 5-6 ml/kg. The animals were pacified by a professional and slept in wooden boxes. The sleeping environment was a quiet and comfortable animal laboratory, and the duration was fixed for 2-4 hours every morning for a total of 8 weeks.

|  | Group OSAHS  (n=6) | control group  (n=6) | Group MAD  (n=6) |
| --- | --- | --- | --- |
| 1/4 Volume | 104.52±28.41 | 141.17±24.45 | 156.53±47.27 |
| 2/4 Volume | 68.34±12.99* | 94.73±110.34 | 103.22±28.49 |
| 3/4 Volume | 50.89±12.85* | 83.36±18.21 | 78.65±15.95 |
| 4/4 Volume | 60.40±19.02* | 97.65±25.37 | 86.95±14.64 |
| Upper Cross-sectional area | 36.67±15.79 | 39.98±3.18 | 45.67±12.88 |
| 1/4 Cross-sectional area | 13.08±2.71* | 19.09±3.32 | 21.07±5.69 |
| 2/4 Cross-sectional area | 10.51±1.69* | 14.44±2.94 | 15.59±3.79 |
| 3/4 Cross-sectional area | 8.77±3.59* | 14.64±3.15 | 14.22±4.31 |
| Lower Cross-sectional area | 12.59±4.02 | 17.73±5.62 | 16.78±4.63 |
| Upper Transverse diameter | 3.37±0.76 | 3.75±0.53 | 4.03±1.17 |
| 1/4 Transverse diameter | 3.12±0.38 | 3.29±0.61 | 3.12±0.62 |
| 2/4 Transverse diameter | 3.32±0.56 | 3.82±1.07 | 3.76±0.30 |
| 3/4 Transverse diameter | 2.86±0.70* | 4.19±0.93 | 4.15±0.82 |
| Lower Transverse diameter | 4.16±0.96 | 5.50±1.27 | 5.04±0.95 |
| Upper Sagittal diameter | 12.23±2.92 | 11.29±3.18 | 13.06±1.31 |
| 1/4 Sagittal diameter | 5.59±1.49* | 7.17±0.35 | 7.72±1.39 |
| 2/4 Sagittal diameter | 3.56±0.70* | 4.82±0.92 | 4.92±1.07 |
| 3/4 Sagittal diameter | 2.99±0.86* | 4.40±0.91 | 4.22±0.76 |
| Lower Sagittal diameter | 4.50±0.96 | 4.92±0.77 | 4.75±0.43 |

* P＜0.05 compare with Group MAD and control group.

**Table S3**. Data of upper airway three-dimension modles in three groups. The volume, cross-sectional area, transverse diameter and sagittal diameter at each level of upper airway, respectively. The results were expressed in mean±SD. Statistically significant differences are indicated by asterisk; *, P<0.05; OSAHS, obstructive sleep apnea-hypopnea syndrome; MAD, mandibular advancement device.

|  | Group OSAHS(n=6) | control group(n=6) | Group MAD(n=6) |
| --- | --- | --- | --- |
|  |
| AHI | 14.75±3.20* | 2.58±1.42 | 4.70±2.35 |
| SaO_2_% | 74.30±8.56* | 86.24±8.98 | 86.35±6.99 |

* P<0.05 compare with group MAD, control group.

**Table S4.** The AHI and SaO_2_% recorded by PSG, respectively. The results were expressed in mean±SD. Statistically significant differences are indicated by asterisk; * P<0.05; OSAHS, obstructive sleep apnea-hypopnea syndrome; MAD, mandibular advancement device; AHI, apnea-hypopnea index; Sao_2_%, oxygen saturation.

| Group | N | HIF-1α/GAPDH |
| --- | --- | --- |
| Group OSAHS | 6 | 1.10±0.22* |
| control group | 6 | 0.48±0.12 |
| Group MAD | 6 | 0.52±0.10 |

* P<0.05 compare with group MAD, control group.

**Table S5.** The expression of HIF-1α. The results were expressed in mean ± SD. Statistically significant differences are indicated by asterisk; *, P <0.05; OSAHS, obstructive sleep apnea-hypopnea syndrome; MAD, mandibular advancement device; HIF-1α, hypoxia-inducible factor-1α; GAPDH, glyceraldehyde-3-phosphate dehydrogenase.


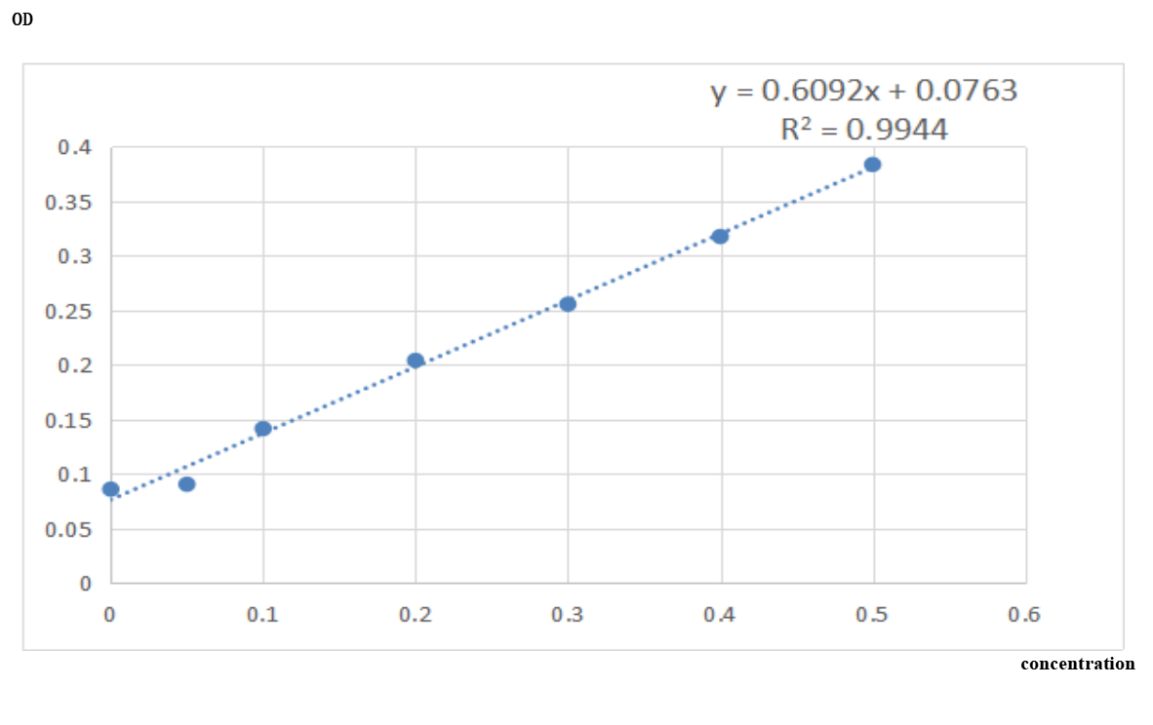


**Figure S6.** Quantitative standard curve of HIF-1α protein in the nucleus of myocardial tissue.

| Group | N | HIF-1α /GAPDH |
| --- | --- | --- |
| Group OSAHS | 6 | 2.17±0.66* |
| control group | 6 | 1.04±0.16 |
| Group MAD | 6 | 1.20±0.32 |

* P<0.05 compare with group MAD and control group.

**Table S7.** The relative expression of HIF-1α mRNA. The results were expressed in mean ± SD. Statistically significant differences are indicated by asterisk; *, P <0.05; OSAHS, obstructive sleep apnea-hypopnea syndrome; MAD, mandibular advancement device; HIF-1α, hypoxia-inducible factor-1α; GAPDH, glyceraldehyde-3-phosphate dehydrogenase.

**
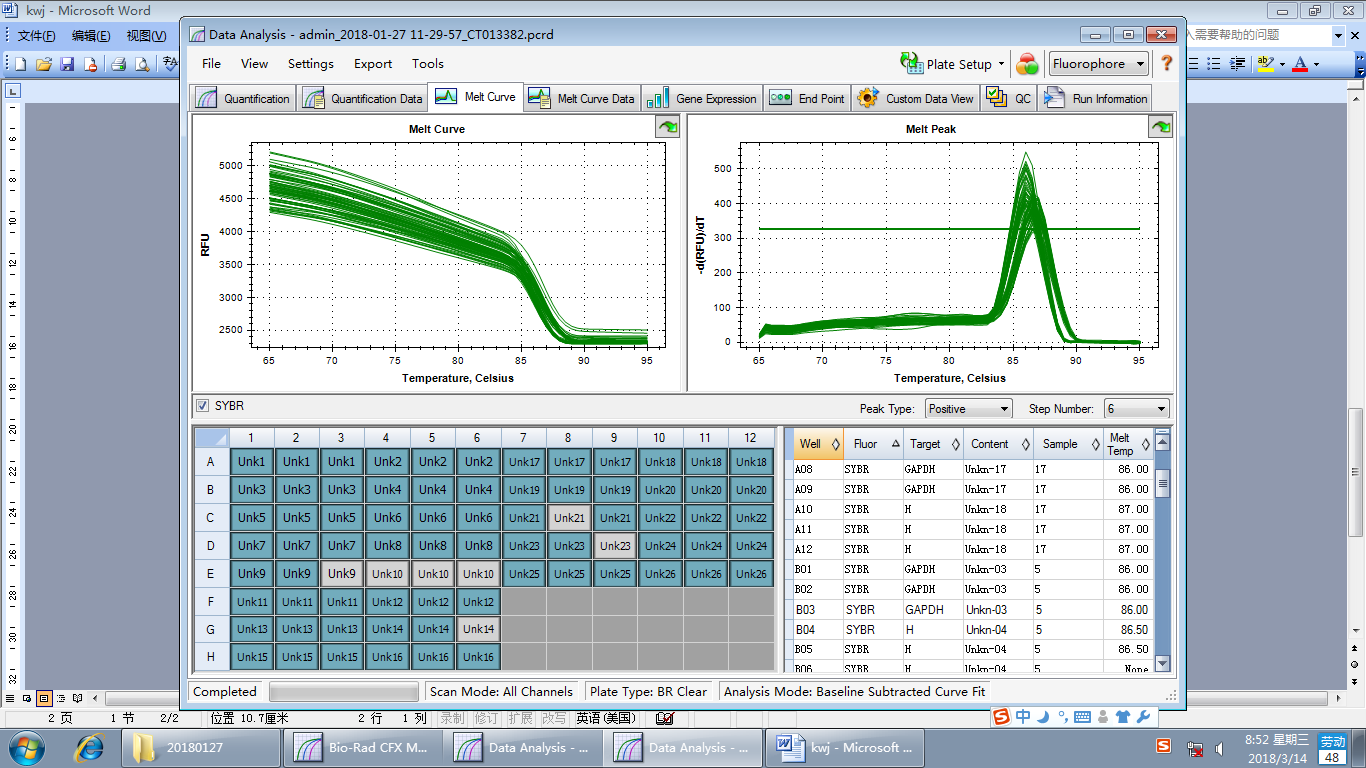
**

**Figure S8.** HIF-1α mRNA dissolution curve in myocardial tissuetissue


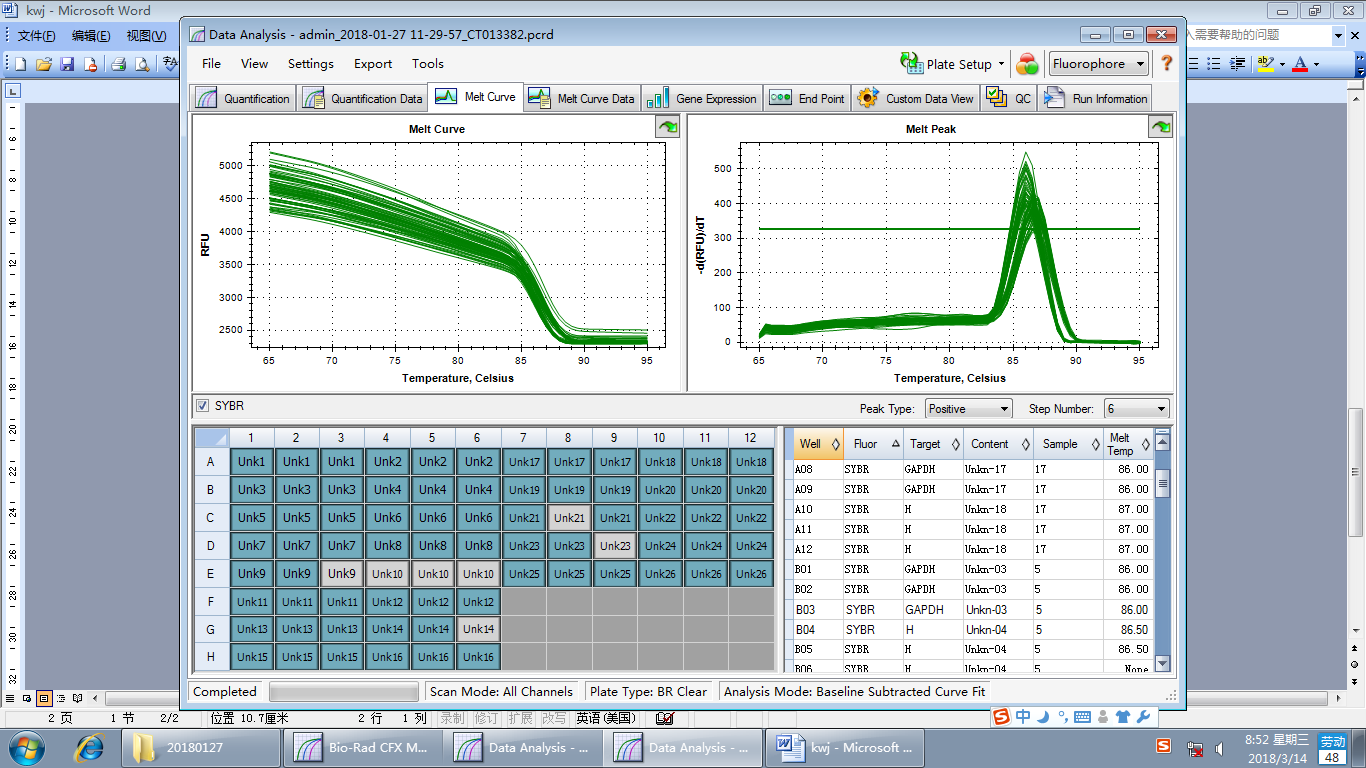


**Figure S9.** The peak value of HIF-1α mRNA dissolution curve in myocardial


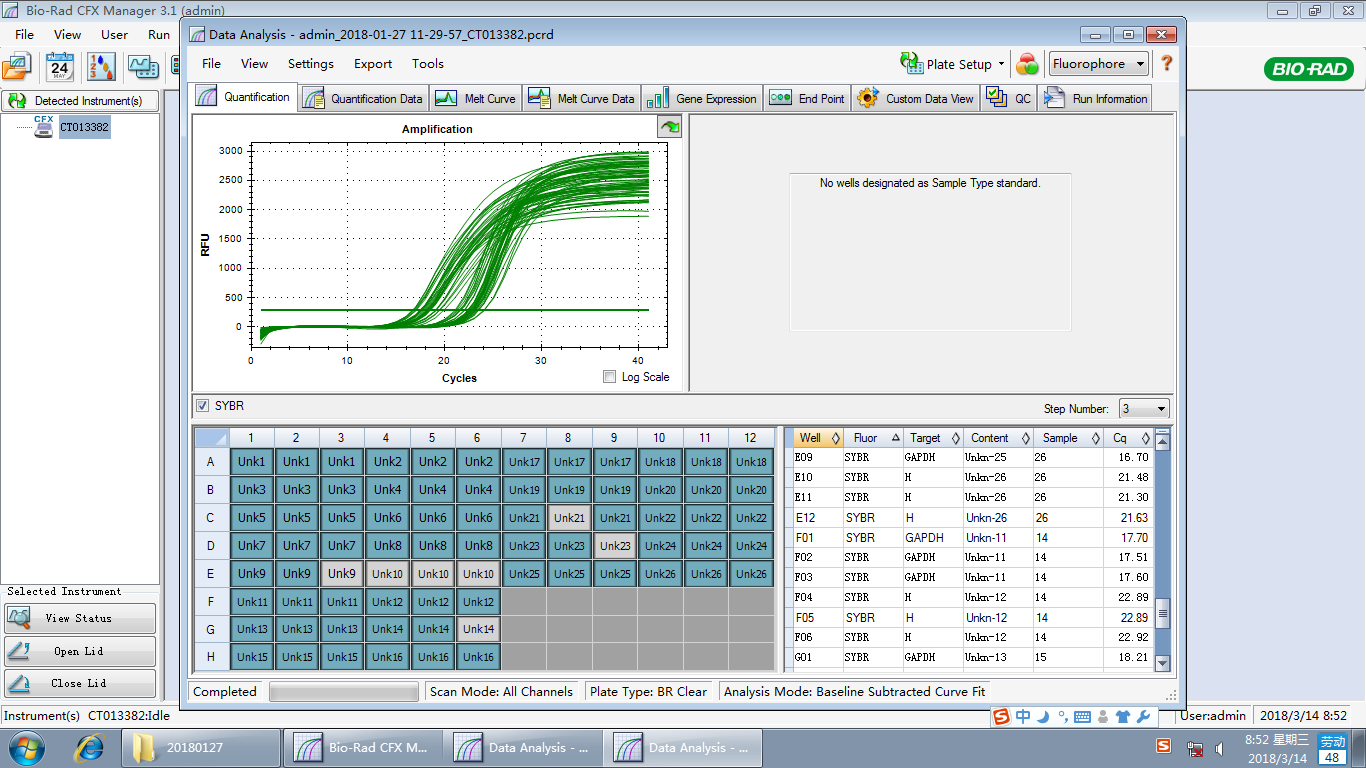


**Figure S10.** HIF-1α mRNA amplification curve in myocardial tissue.

|  | Group OSAHS  (n=6) | control group  (n=6) | Group MAD  (n=6) |
| --- | --- | --- | --- |
| EPO (pg/ ml) | 234.27±132.17* | 75.30±7.11 | 78.69±10.12 |
| VEGF(pg/ ml) | 451.85±31.10* | 337.46±17.98 | 374.48±22.35 |

* P<0.05 compare with Group MAD and control group.

**Table S11.** Concentration of EPO and VEGF. The results were expressed in mean±SD. Statistically significant differences are indicated by asterisk; *, P<0.05; OSAHS, obstructive sleep apnoea-hypopnea syndrome; MAD, mandibular advancement device; EPO, erythropoietin; VEGF, vascular endothelial growth factor.

**
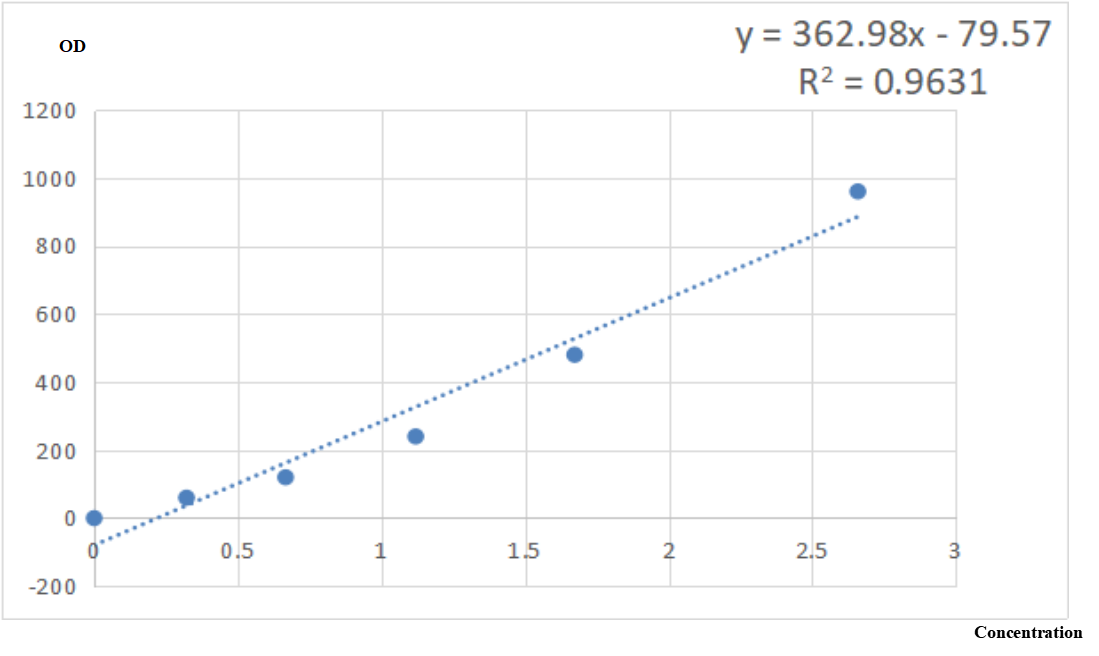
**

**Figure S12.** The standard EPO curve of myocardial tissue.


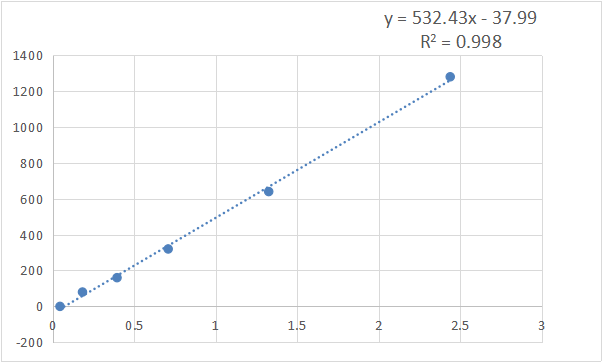


**Figure S13.** The standard VEGF curve of myocardial tissue.


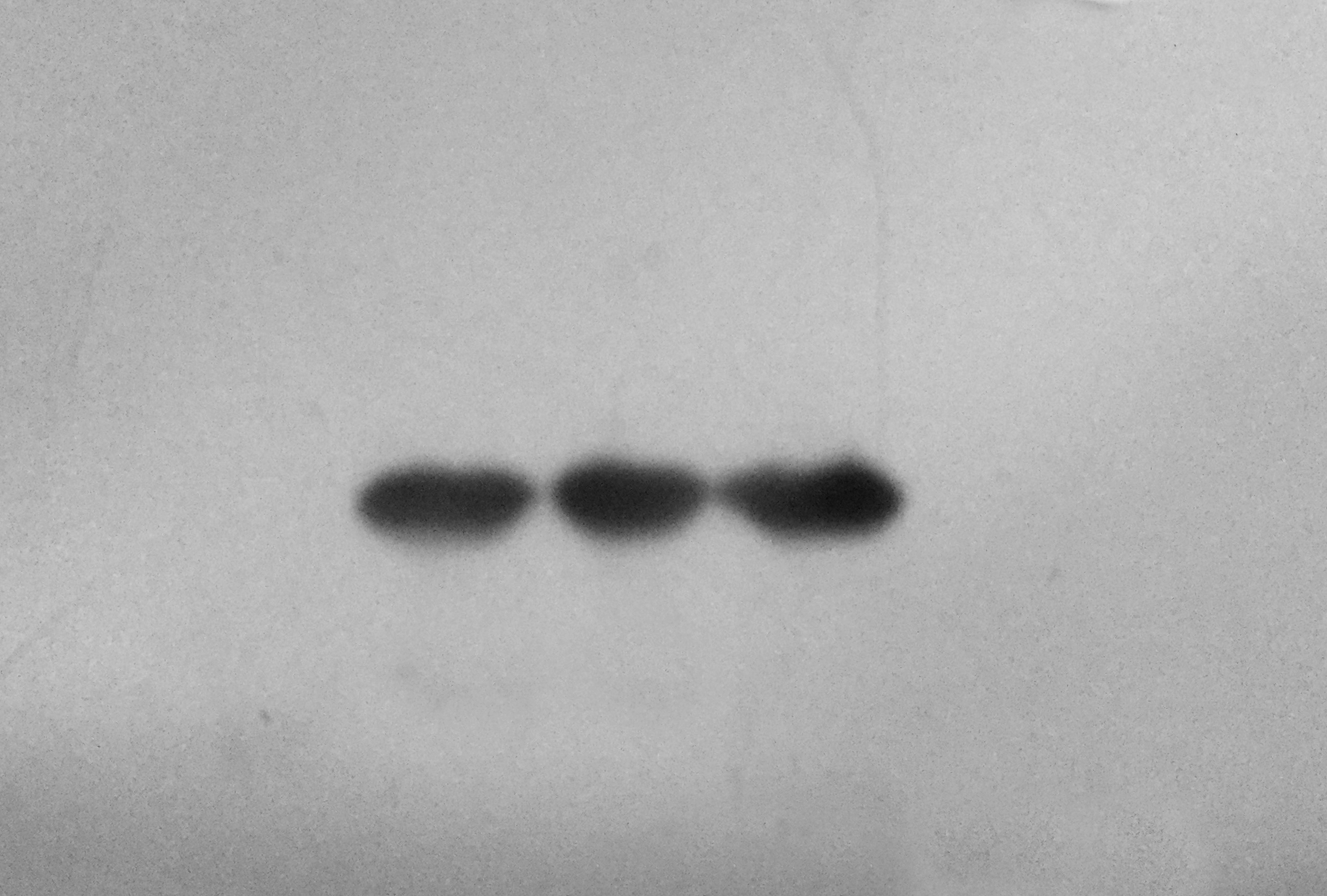

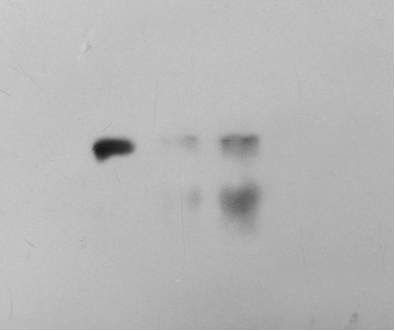


OSAHS Control MAD

140KDa

100KDa

80KDa

HIF-1α（90KDa）

60KDa

50KDa

OSAHS Control MAD

40KDa

GAPDH（36KDa）

30KDa

Fig. S14. Uncropped images of Figure 7 a
